# Comparative Analysis of Different ELISA Methods for the Serodiagnosis of *Przhevalskiana silenus* Infestation in Goats

**DOI:** 10.3390/vetsci10060396

**Published:** 2023-06-16

**Authors:** Sadia Liaqat, Mazhar Qayyum, Figen Celik, Sami Simsek, Faheem Ahmad, Xiaocheng Zhang, Haroon Ahmed, Jianping Cao

**Affiliations:** 1 Department of Zoology, PMAS Arid Agriculture University, Rawalpindi 46300, Pakistan; 2 Department of Parasitology, Faculty of Veterinary Medicine, University of Firat, 23119 Elazig, Turkey; 3 Department of Biosciences, COMSTS University Islamabad (CUI), Islamabad 46400, Pakistan; 4 Key Laboratory of Parasite and Vector Biology, National Health Commission of the People’s Republic of China, Shanghai 200025, China; 5 National Institute of Parasitic Diseases, Chinese Center for Disease Control and Prevention (Chinese Center for Tropical Diseases Research), Shanghai 200025, China; 6 World Health Organization Collaborating Center for Tropical Diseases, Shanghai 200025, China; 7 The School of Global Health, Chinese Center for Tropical Diseases Research, Shanghai Jiao Tong University School of Medicine, Shanghai 200024, China

**Keywords:** hypodermosis, myiasis, immunodiagnosis, indirect ELISA, *Przhevalskiana silenus*, warble fly, goat warble fly infestation, Pakistan

## Abstract

**Simple Summary:**

Warble fly is a globally important pest of domesticated and wild animals in the northern hemisphere. Goat warble fly infestation (GWFI), triggered by the obligatory parasite *Przhevalskiana silenus* larvae, has detrimental consequences for livestock. GWFI is usually not accurately detected by the traditional methods of detection. This study aimed to identify the best diagnostic method for the early detection of GWFI by comparing three enzyme-linked immunosorbent assay (ELISA) methods using Hypoderma purified antigen (Hypodermin C), the crude antigen of *P. silenus*, and a commercially available hypodermosis ELISA kit, in the Pothwar region of Punjab, Pakistan. The sampling was carried out in 1000 goats, randomly selected from four districts (Rawalpindi, Attock, Jhelum, and Attock) of the Pothwar region, Punjab Pakistan. The seroprevalence, determined via ELISA with the crude *P. silenus* antigen, hypodermin C (HyC) antigen, and the commercial ELISA kit, was 17%, 15.5%, and 18.6%, respectively. Cohen’s Kappa test confirmed that all the three assays could be employed for the early detection of GWFI, while an indirect crude *P. silenus* antigen ELISA was more sensitive and accurate.

**Abstract:**

*Przhevalskiana silenus* (warble fly) grubs cause myiasis in goats, in mountainous and semi-mountainous areas and different regions in Pakistan, and cause substantial losses to livestock. The palpation method for detecting warble flies generally neglects the infestation intensity; therefore, the development of a reliable and efficient diagnostic technique is extremely necessary. This study compared three indirect enzyme-linked immunosorbent assay (ELISA) methods for detecting anti-*P. silenus* antibodies using the hypodermin C (HyC) purified from Hypoderma spp. Larvae collected in cattle (local isolate, Microbiology Laboratory, PMAS-Arid Agriculture University, Rawalpindi), the crude antigen from the first instar stage of *P. silenus*, and a commercial Bovine Hypodermosis Antibody ELISA kit (IDEXX Laboratory), for accurately estimating the seroprevalence of goat warble fly infestation (GWFI) in the Pothwar plateau, Punjab, Pakistan. The ELISA with the crude antigen of *P. silenus* proved very sensitive and specific, 91% and 93%, respectively. The optical density exhibited a monthly variation, and the antibody titer began increasing from June, continually increased from July to December, and gradually decreased thereafter until March. The study confirmed the endemic status of GWFI in the Pothwar region and identified that ELISA based on the crude antigen of *P. silenus* was a more sensitive and specific immunodiagnostic method for determining seroprevalence, and could be employed for initiating nationwide eradication campaigns.

## 1. Introduction

Myiasis in goats, caused by the grubs of *Przhevalskiana silenus* (Diptera, Oestridae), are obligatory parasites that feed on the living tissues of hosts for a variable period. This infection reduces host physiological functions and affects livestock and the leather industry by degrading the hide of infested animals [1]. The reduced productivity and depreciation of the carcass further worsens protein deficiency for human consumption [2]. The infection has a colossal economic impact worldwide and especially in Pakistan, where leather and leather products generate the biggest overseas profit. The losses due to this menace cannot be calculated due to a number of factors, while the hide damage was the most important consequence of the infestation, resulting in the low price on the account of the holes formed by the warble fly. This damage to the livestock industry affected the country’s economic growth, which proved inevitable, as this infestation was not detected at an earlier phase [3].

In endemic areas, an accurate diagnosis of myiasis holds much significance for the initiation of control strategies leading toward eradication policies. There are numerous diagnostic techniques designed for the diagnosis of goat warble fly infestation (GWFI). The traditional diagnostic approach relied entirely on the palpation in the flank and back region of the infested host, where nodules formed at a later phase of the infection. This physio clinical observation is usually inaccurate in detecting infestation at the first and second instar (L1, L2) stages, as they are occult under the host’s skin. Thus, there is a dire need for an efficient diagnostic method that assists the diagnosis and controls the infestation at the herd level in proper time (L1), and its prophylactic management to overcome economic impacts [4,5]. The application of molecular and serological tools can provide extremely important analytical data for the diagnosis and control of a variety of parasitic diseases [6,7].

In past, in vivo and in vitro studies reported the specific and nonspecific host’s immune responses to infestation by myiasis-causing larvae, and advances were made in immunological approaches by parasite the against their host. The broad investigations were conducted on the contribution of the antigens of myiasis-causing larvae as modulators of the host immune response. For the diagnostic and development of vaccination strategies, the practical implications of immunological knowledge are of great significance. An alternative to chemical treatments, the developing environmentally sustainable control methods proved more practical. Immunological methods for the detection of myiasis allows for reliable, easy, and cost-effective diagnosis of living animals, thus scheduling a timely diagnosis. Nevertheless, the data regarding the host immune response to myiasis-causing larvae are fragmentary compared to other ectoparasites [8].

An enzyme-linked immunosorbant assay (ELISA) is extensively practiced for the detection of hypodermosis because of its accuracy. The detection of the circulating antibodies through ELISA has become renown and facilitated the eradication of this menace from various regions of the world [9,10]. The similarities in the immune response to bovine hypodermosis caused by the larvae of *Hypoderma* spp and other myiasis-causing larvae are considered to be useful for the immunodiagnosis of hypodermosis in various mammals [11,12,13,14,15,16,17,18]. However, reliable and sensitive immunological methods, such as ELISA, for the diagnosis of hypodermosis, is strongly recommended [12,19,20]. Furthermore, a previous study confirmed that ELISA kits prepared for the detection of bovine hypodermosis can be applied for detecting antibodies in goats infested with warble flies [21]. A recent study verified that the L1 antigen of *Przhevalskiana* sp can be employed for the diagnosis of anti-*Przhevalskiana* sp antibodies using competitive ELISA [22]. Recently reported an optimized indirect ELISA using recombinant HyC extracted from *P. silenus* for the accurate and timely diagnosis of GWFI. The optimized indirect ELISA became a verified immunodiagnostic procedure for the mass surveillance of this infestation [23].

In the Pothwar plateau, goat farming is a widespread business not only for milk, meat, skin, and cheese production, but also because this species can exploit massive areas of peripheral lands that is unfavorable for crop cultivation and rearing other species. The prevalence of GWFI has been reported from many parts of Pakistan, whereas direct and indirect economic impacts due to this infestation have not been precisely indicated. So far, the position of myiasis in goats by *Przhevalskiana* spp in the country has not been explored in detail, as the diagnosis of infestation was based merely on clinical examination. The present study aimed to compare three ELISA methods for the accurate detection of anti-*P. silenus* antibodies using the HyC antigen, crude antigen extracted from *P. silenus*, and a commercially available hypodermosis ELISA kit for estimating the seroprevalence of GWFI in the Pothwar plateau of Punjab, Pakistan.

## 2. Materials and Methods

### 2.1. Study Area

The warble fly is endemic to semi-hilly, riverine, and mountainous areas of Pakistan [24]; that is why the serological investigation was carried out on goats in the Pothwar plateau, Punjab, Pakistan. The Pothwar plateau is located north of the Punjab province, covering an area of 13,000 sq km, representing 2.9% of the country. The region is bounded by the river Jhelum and Indus on the east and west, while the Kala Chita, Salt Range, and Murree Hills are on northern and southern sides. The plateau serves as suitable pasture land with its plane to moderately undulating topography, separated by hills and valleys that are ideal for the growth and development of warble fly. This region includes four districts i.e., Jhelum (233 m), Attock (355 m), Chakwal (498 m) and Rawalpindi (508 m) (including Islamabad, the Capital territory), with average temperature of 22.5 °C [25]. This region is a rain-fed zone with a suitable temperature, having a large number of livestock that further assist the living conditions of the warble fly.

### 2.2. Experimental Design

Blood samples were collected from 1000 goats that were randomly selected (25 per month per district) from four district (Attock, Chakwal, Jhelum, and Rawalpindi), of the Pothwar region, Punjab, Pakistan. Ten sites belonging to three different categories, namely, farms, houses, and veterinary hospitals, from each of the four districts were selected for sampling during the study period, which lasted from June 2018 to March 2019. Blood was drawn from the jugular vein and placed in 10 mL non-Ethylenediaminetetraacetic acid (EDTA) tubes and centrifuged to separate the sera. The sera were then allocated in Eppendorf tubes (1 mL) and stored for further analysis at −20 °C. The samples of the positive sera were collected from goats infested with warble fly in the Pothwar region of Pakistan, while the negative sera samples were obtained from the Lancaster Goats, Scotland.

### 2.3. Larvae Collection and Preservation

An indirect ELISA was performed for detecting anti-*P. silenus* antibodies based on the crude antigen extract obtained from the L1 of *P. silenus*. This sampling was carried out from 100 *P. silenus*-infested goats, from a slaughter house in Saddar, Rawalpindi, from June to September. The L1 was extracted from the dissection of hypodermal tissues of the slaughtered goats, and after washing with normal saline, they were preserved in a phosphate buffer saline solution (PBS) at −20 °C. The larval identification was achieved based on their morphological features [26,27].

### 2.4. Crude Antigen of P. silenus

The crude antigen of *P. silenus* was prepared by adding 10 L1 per 5 mL (0.1 M) tris-hydrochloric acid (pH = 7.5), were then grinded in tissue homogenizer, using five cycles of 1 min at 14,000 rpm. The homogenized material was placed in an ice bath to stop the temperature rise that could otherwise denature the proteins by activating the enzymatic processes between the cycles. The homogenate was centrifuged at 10,000 rpm for 5 min, followed by the separation of the supernatant in aliquots, and kept at −20 °C.

### 2.5. Determination of Protein Concentration

The Pierce Bicinchoninic Acid (BCA) technique was used for the estimation of protein concentration in the antigen.

### 2.6. HyC Antigen

Hypodermin C was purified from *Hypoderma lineatum* first instars obtained from cattle slaughtered in local slaughterhouses, Rawalpindi [28].

### 2.7. Indirect ELISA (Crude Antigen of P. silenus)

An indirect ELISA was performed using a simple 96-well microtitration plate (Delta labs, Lahore, Pakistan). The plate was coated with 100 μL (5 μL/mL) of the crude antigen of *P. silenus* and incubated at 37 °C, overnight. Then 200 μL of the dilution buffer PTL (1.2 g skim milk + 60 mL of PBS-Tween) was dispensed in each well and blocked at 37 °C for 30 min. The plate was then washed 4 times (3 times with 100 μL + 1 time with 200 μL PT). Of the diluted sera, 100 μL (1:10) were added in duplicate. The negative and positive sera (standards) were also added in same dilutions and incubated for 1 h. The plate was then washed with PT (1 time 200 μL + 3 times with 100 μL), followed by an addition of 50 μL (1:10,000) of rabbit anti-goat IgG (H + L) (Southern Biotech, Birmingham, USA) immuno-conjugate (horseradish peroxidase conjugate). Then, the incubation was set at 37 °C for 1 h and washed again (1 stretch of 200 μL and 2 stretches of 100 μL PT + 2 stretches 100 μL PBS). The TMB substrate (3,3′,5,5′-tertramethylbenzidine) was then poured in each well and placed in the dark for 5–20 min, followed by the addition of 100 μL, 3 N sulfuric acid, then the reaction stopped. The optimal density (OD) was measured at 492 nm using a FLUOstar OPTIMAspectrophotometer (BMG Lab TECH Ltd., Cary, FL, USA). The following formulae were used for calculating the specificity and sensitivity (Equations (1) and (2)):

Diagnostic specificity (DSP) = True negative/(True negative + false positive)(1)

Diagnostic sensitivity (DSN) = True positive/(True positive + false negative)(2)

### 2.8. Indirect ELISA (HyC Antigen)

For anti-*P. silenus* antibody detection based on the HyC antigen, an indirect ELISA was standardized using 100 μL (2 μL/mL) HyC antigen and 50 μL (1:10,000) immuno-conjugate (horseradish-peroxidase conjugated) rabbit anti-bovine Ig G (H + L) (Geneway: GWBC158CO) in each well. The ELISA protocol, with minor modifications, was used for the bioassay [9,28]. The OD was measured at a wavelength of 492 nm using a FLUOstar OPTIMA spectrophotometer. The DSN and DSP were also estimated, as mentioned in Section 2.6.

### 2.9. Determination of the Cut-Off Value

The following formula (Equation (3)) was used for calculating the cut-off values:

Cut-off value = Mean value of negative control panel ± 2 Standard Deviation(3)

The sample, with an OD value less than the cut-off value, was regarded as negative, while those more than the cut-off value was considered positive.

### 2.10. ELISA Using Commercial Kits

A Bovine Hypodermosis Antibody ELISA kit (IDEXX Laboratories, Inc., Westbrook, ME, USA) was found commercially. The ELISA protocol was given by the manufacturer of the test kit [29,30]. A wavelength of 450 nm was selected on FLUOstar OPTIMA spectrophotometer to record the OD values of the control and samples, and the formulae used to further calculate the results are given below (Equations (4)–(6)).

#### 2.10.1. Mean (OD) Negative Control (NCx^−^)

The following formula is used to calculate NCx^−^;

where,

NC1 = OD for negative control 1 NC2 = OD for negative control 2 NCx^−^ = NC1 A (450) + NC2 A (450)/2 (4)

#### 2.10.2. Mean (OD) Positive Control (PCx^−^)

The following formula is used to calculate PCx^−^;

where,

PC1 = OD for positive control 1 PC2 = OD for positive control 2 PCx^−^ = PC1 A (450) + PC2 A (450)/2 (5)

#### 2.10.3. Calculation of Seropositivity (S/P%)

The following formula is used to calculate S/P%;

where,

Sample = OD value of the samples S/P% = [Sample A450 − NCx^−^/(PCx^−^) − (NCx^−^)] × 100 (6)

#### 2.10.4. Result Interpretation

The samples with seropositivity (S/P%) more than 55% were regarded as positive, while those with S/P% greater than 45%, but less than 55%, were suspected to be positive, and the samples with S/P% less than 45% were regarded as negative for *P. silenus*.

### 2.11. Validation of ELISA

The validation of ELISA was performed on two hundred sera samples taken from *P. silenus*-infested goats with prominent nodules. This sampling was carried out in the month of December, from the slaughter houses of the study area.

### 2.12. Statistical Analysis

The district- and month-wise seroprevalence was statistically evaluated by Pearson’s chi-square (χ^2^) test, and the variables will be considered statistically significant at *p* < 0.05. The inter-rater reliability between the different diagnostic methods were analyzed via Cohen’s Kappa test, using SPSS, version 18.

## 3. Results

For the serological study, a commercial ELISA Kit, the crude antigen of *P. silenus*, and HyC antigen ELISA methods were compared for the detection of GWFI at an early stage.

### 3.1. Development and Validation of the Crude Antigen of P. silenus ELISA

An indirect ELISA, using the crude antigen of *P. silenus* larvae (L1) was performed on one thousand goat sera obtained from various districts of the Pothwar region, Punjab, Pakistan. Two hundred warble-fly-infested goats were selected for ELISA validation. The blood sampling was performed in the month of December from goats with prominent nodules, and an indirect ELISA was performed. The developed crude antigen of *P. silenus* ELISA showed a sensitivity and specificity of 98%.

### 3.2. Comparison of Serological Bioassays

The cut-off values of the indirect ELISA tests performed using the crude antigen obtained from *P. silenus* larvae and HyC antigen were 0.38 (0.224 ± 0.156) and 0.226 (0.153 ± 0.073), respectively. The sensitivity of the ELISA tests using the crude antigen, HyC antigen, and the commercial kit was determined to be 91%, 88%, and 90%, respectively, while the specificity was determined to be 93%, 93%, and 90%, respectively. Cohen’s Kappa test confirmed that the findings of all three assays moderately vary and could be employed for the early detection of GWFI. (Table 1).

### 3.3. District-Wise Seroprevalence of GWFI Based on Serological Analyses with a Commercial ELISA Kit, and ELISA Performed Using the Crude Antigen and HyC Antigen

The present study determined the seroprevalence of GWFI in the four districts of the Pothwar region of Pakistan, with different climatic conditions and geographical boundaries. The mean seroprevalence determined with the commercial kit, crude antigen of *P. silenus*, and HyC antigen ELISA was 18.5% (185/1000), 17% (170/1000), and 15.5% (155/1000), respectively, showing a significant difference (*p* < 0.05). The study revealed that the seroprevalence was higher in the Jhelum district, being 30.8%, 28%, and 25.6% as determined via an ELISA using the commercial kit, crude antigen of *P. silenus*, and HyC antigen ELISA, respectively. In the Rawalpindi district, the seroprevalence was determined to be 24.4%, 23.6%, and 22.4%, respectively; the seroprevalence was low in Chakwal district (13%, 9.2%, and 8.8%, respectively) and the lowest in the Attock (6%, 7.2%, and 5.2%, respectively) district (Table 2).

### 3.4. Variations in OD

**Figure 1:**
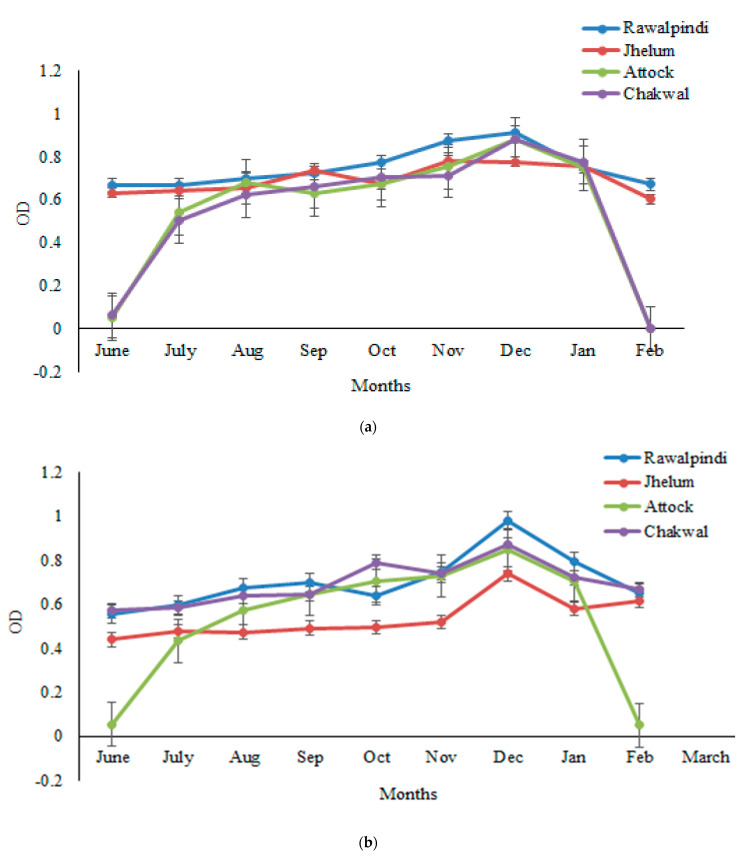

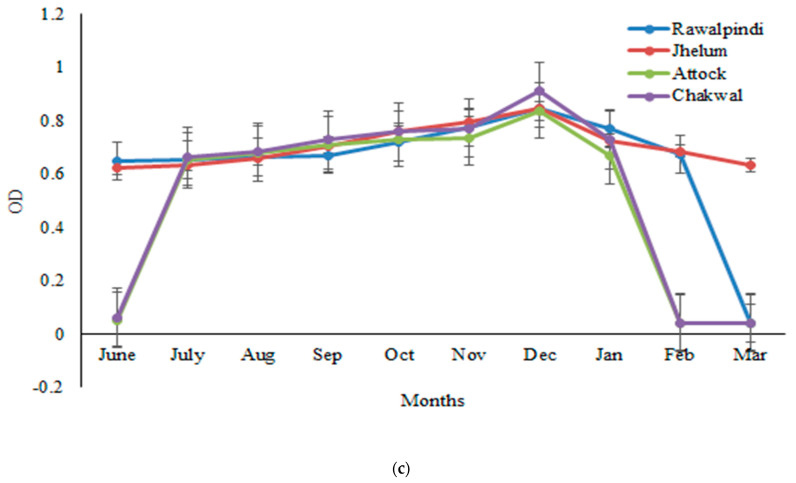
Monthly variations in the mean OD values determined following ELISA with (**a**) HyC antigen, (**b**) a commercial ELISA kit, and (**c**) the crude antigen of *P. silenus*, from the positive sera of goats in the Pothwar region of Punjab, Pakistan.

The sera samples obtained from one thousand goats randomly selected from four districts (Rawalpindi, Attock, Jhelum, and Chakwal) were analyzed for GWFI through ELISA, and the OD was determined at a wavelength of 492 nm and 450 nm. The findings revealed that the OD values exhibited a monthly variation. The antibody titer started increasing from June and increased continually from July to December, but decreased gradually thereafter until March. The peak antibody titer was found in the months of November–December, followed by a decrease afterward. The highest seroprevalence was observed in winter months, i.e., November to December, and the lowest in March. The seroprevalence was 47%, 45%, and 44% in December by crude *P. silenus* antigen ELISA, HyC antigen ELISA and commercial kit, respectively. The highest seroprevalence was observed in the winter months, i.e., November to December and lowest in the March, whereas a low antibody titer was found in June and July. Further, antibodies were not observed in the sera samples of goats before the month of June. Statistically, the month-wise analysis of the data revealed significant differences (*p* < 0.05) in the seroprevalence (Figure 1a–c).

## 4. Discussion

*P. silenus*, a Dipterian fly, belongs to the family Oestridae; is responsible for heavy economical losses in livestock and the leather industry due to perforated skin and hides [1]. The warble fly is endemic to hilly and semi-hilly areas of the world. In spite of the broad distribution and detrimental losses, the farmers and veterinarians still underestimated the infestation. The traditional palpation method detects GWFI in winter (November–February) when nodules start appearing on the skin of infested animals, which underestimated the disease and much damage has been done to the animal. Therefore, the development of a reliable and efficient diagnostic technique is extremely necessary [4,5]. Various serological assays are performed for the early detection of infestation in warble-fly-endemic areas. ELISA proved a valuable diagnostic tool for epidemiological surveillance studies worldwide. An earlier study has employed ELISA, based on HyC antigen, for detecting antibodies from goat sera, thus verifying the cross-reactivity [18]. It has also been demonstrated in another study that ELISA based on recombinant HyC is convenient for detecting anti-*Hypoderma* spp. antibodies at an early stage of infection [8]. The commercial ELISA kit (Bovine serum Hypodermosis) is considered a simple and rapid diagnostic tool for the serodiagnosis of GWFI showing cross-reactivity (*H. lineatum* antigen and anti-*P. silenus* antibody) [31,32,33]. The HyC is a common enzyme in several hypoderma spp. has been widely used in the serological diagnosis of hypodermosis by other species [34].

The present study, is the first to perform an ELISA using the crude antigen of *P. silenus* for the serodiagnosis of GWFI in the Pothwar region of Punjab, Pakistan, and the results were compared with HyC antigen ELISA and commercial ELISA kit. It has been confirmed that an indirect ELISA helps diagnose GWFI at an earlier stage, because at this stage, some animals have antibodies in their blood, but they do not show any nodules; whereas, the physio clinical examination diagnoses the infestation at the later stage. When nodules start appearing on an animal body and much damage has been done to the host. The sensitivity and specificity of the indirect crude antigen (*P. silenus*) ELISA are higher than those of HyC antigen ELISA and the commercial kit. Statistical analysis, with the Cohen’s Kappa, test confirmed that the results of all the three immunodiagnostic methods varied moderately, which confirmed that all the three serological methods could be employed for the early detection of GWFI. However, of the three methods, the developed indirect ELISA method based on the crude antigen of *P. silenus* proved to be the most accurate and had a higher sensitivity and specificity. Contrary to that, a previous study reported that a commercial ELISA kit is highly accurate and has a high sensitivity and specificity of 92.2% and 98.1%, respectively [35]. An earlier study confirmed that the commercial ELISA kit proved useful in providing baseline information for the control of this economically important parasite [20]. The developed indirect ELISA using the crude *P. silenus* antigen is more sensitive and cost-effective, whereas the commercial kit previously used is expensive and cannot be procured by the farmers of developing countries.

The overall district-wise seroprevalence of GWFI was also studied across the districts of Rawalpindi, Jhelum, Attock, and Chakwal. The seroprevalence determined by ELISA with the crude *P. silenus* antigen, HyC antigen, and the commercial ELISA kit was 17%, 15.5%, and 18.6%, respectively. The results of descriptive analysis revealed that the seroprevalence of GWFI was higher in the Jhelum and Rawalpindi districts, but low in Chakwal and Attock. The differences among the seroprevalence of GWFI across the districts were determined to be significant (*p* < 0.05). Similarly, the goats of the Khoshab and Chakwal districts of the Punjab province were examined (clinically) for the prevalence of warble. The larvae were collected from the infested goats and identified as *P. silenus*. The results showed that the prevalence of GWFI was 17.8% (89/500) [36]. This study’s results correlate with the previous findings in Pakistan, showing 25% [37], 24.9% [27], in Iran 7% to 18.9% [38], and Jordan 10% [39], GWFI reported cases. Contrary to that, in the Rakhi Gauge and Rakhi Manu area of Pakistan, 40% and 41% goats were infested with warble fly [40]. These results contradict the present research results, which might be due to the use of antiparasitic drugs and different diagnostic method (hand palpation) in the study areas. The seroprevalence of warble fly infestation from the four districts of the Pothwar region were estimated for the first time, while all previous studies were based on traditional diagnostic techniques. Earlier sero-epidemiological studies were conducted on goat warble fly to observe the prevalence of hypodermosis in district Ziarat, northern upland of Balochistan, Pakistan. In that study, the seroprevalence of GWFI was 64.16% according to the results of a commercial ELISA kit [20]. The prevalence of GWFI is affected by variations in altitude and other environmental factors, including humidity, temperature, season, and rainfall received [41], as well as other factors such as the management system, specificity, grazing patterns, and the use of insecticides [28].

The seroprevalence of GWFI was monitored in a monthly manner during the study period (June–March), and revealed significant differences (*p* < 0.05). The sera obtained from the goats randomly selected from the study area were analyzed for GWFI through ELISA, and the OD was determined at a wavelength of 492 nm and 450 nm. The findings revealed that the OD values exhibited a monthly variation. The antibody titer started increasing from June, and increased continually from July to December, but decreased gradually thereafter unit March. The highest seroprevalence was observed in the winter months, i.e., November to December, and the lowest in March. However, hand palpation showed no infestation from March to September, as the larvae were at a subclinical phase, which were only detected through serological tests. The results of ELISA tests revealed that the antibody titer in the blood samples of the animals infested with warble flies increased from June to September. The antibodies were not seen before the month of June as in these months, the fly was in a different developmental stage. In Pakistan, the life cycle of myiasis starts from April and L1 starts appearing from June to September, making this period ideal for serodiagonsis by ELISA as animals show a higher antibody titer. The highest seroprevalence was observed in the winter months, i.e., November to December, and the lowest in March. These observations are in agreement with the decline in the OD values which depends on the maturation of the instars [42]. A recent study, conducted on the antigen capture ELISA revealed a quick fall in the antigen level following the arrival of grubs at the back [10]. The findings confirmed that the nodules start disappearing in January in endemic areas with GWFI in Pakistan [43]. The present study confirmed the higher prevalence from November to December, which is almost similar to the previous study [44], based on seasonal variations in prevalence, with higher values from November to February, and maximum (94.4%) in November. Earlier, in the Balochistan province, the month-wise prevalence under an agriculture linkage program (ALP) project, also revealed the highest prevalence during the months of November and January [45]. The present findings also coincide with a previous study showing a significant difference in the seroprevalence amongst the months, which demonstrated the highest prevalence (92%) during November and lowest (20%) during the month of March, and also concluded that the best treatment time for GWFI is during June to July [21]. The present findings are in agreement with previous studies, with the lowest month-wise prevalence in July and the highest in the month of December [43].

## 5. Conclusions

In conclusion, the present study identified the endemic status of GWFI and is accomplished as an alarming threat to animal health in the Pothwar region of Pakistan. The study confirmed that all three ELISA bioassays (crude *P. silenus* antigen, HyC antigen, and the commercial ELISA kit) could be employed for the early detection of GWFI, and showed better results as compared to traditional palpation method. During the study, an ELISA with the crude antigen of *P. silenus* was developed that proved to be a more rapid, sensitive, and specific immunodiagnostic method for determining the seroprevalence of GWFI. In a comparative study of HyC antigen ELISA, anti-bovine immunoconjugate was used that showed better results, but it is suggested that for more specificity, anti-goat immunoconjugate could be used. This is a cross-sectional study where each animal was sampled once, so different animals were sampled in each month. Taking that in consideration, a longitudinal study that follows the same animals over time will be performed in future to determine the monthly variation in OD. The findings highlight that the developed ELISA should be used in other parts of Pakistan to find the exact status of this infestation. Furthermore, this strategy can be employed for initiating nationwide GWFI eradication campaigns.

## Figures and Tables

**Table 1:** Sensitivity and specificity of the ELISA tests performed based on the HyC antigen, crude antigen from *P. silenus*, and a commercial hypodermosis ELISA kit.

	HyC-Based ELISA	Sensitivity (%)	Specificity (%)	Crude Antigen *P. silenus*-Based ELISA	Sensitivity (%)	Specificity (%)	Commercial ELISA Kit	Sensitivity (%)	Specificity (%)
True positive	96	88	93	102	91	93	99	90	90
False positive	59			67			87		
True negative	832			821			803		
False negative	13			10			11		
Total	1000			1000			1000		

**Table 2:** Overall seroprevalence of GWFI from four districts of the Pothwar region, Punjab, Pakistan, determined via an ELISA based on the HyC antigen, *P. silenus* crude antigen, and a commercial ELISA kit.

ELISA Based on	Number of Animals	Districts				*p*-Values
		Rawalpindi	Attock	Chakwal	Jhelum	
	Total	250	250	250	250	
HyC antigen	Infested	56	13	22	64	F = 58
	Non-infested	194	237	228	186	df = 3
	Percentage (%)	22.4	5.2	8.8	25.6	*p* < 0.05
Commerical kit	Infested	61	15	32	77	F = 64
	Non-infested	189	235	218	173	df = 3
	Percentage (%)	24.4	6.0	13	30.8	*p* < 0.05
*P. silenus* crude antigen	Infested	59	18	23	70	F = 56
	Non-infested	191	232	227	180	df = 3
	Percentage (%)	23.6	7.2	9.2	28	*p* < 0.05

χ^2^ Test: HyC antigen ELISA F = 58, degree of freedom (df) = 3, (*p* < 0.05). χ^2^ Test: commercial kit ELISA F = 64, df = 3, (*p* < 0.05). χ^2^ Test: *P. silenus* crude antigen ELISA F = 56, df = 3, (*p* < 0.05).

## Data Availability

Not applicable.
